# Cobalt- and Copper-Based *Chemiresistors* for Low Concentration Methane Detection, a Comparison Study

**DOI:** 10.3390/gels8110721

**Published:** 2022-11-08

**Authors:** Paul Chesler, Cristian Hornoiu, Mihai Anastasescu, Jose Maria Calderon-Moreno, Marin Gheorghe, Mariuca Gartner

**Affiliations:** 1 “Ilie Murgulescu” Institute of Physical Chemistry—Romanian Academy, Splaiul Independentei 202, 060021 Bucharest, Romania; 2 NANOM MEMS SRL, Strada George Cosbuc 9, Rasnov, 505400 Brașov, Romania

**Keywords:** sol–gel, thin films, alumina wafer, IDE, lab conditions, 5 ppm CH_4_, low-cost/eco-friendly, cross-sensitivity/humidity test

## Abstract

Methane is a colorless/odorless major greenhouse effect gas, which can explode when it accumulates at concentrations above 50,000 ppm. Its detection cannot be performed without specialized equipment, namely sensing devices. A series of MOX sensors (*chemiresistors* type), with CoO and CuO sensitive films were obtained using an eco-friendly and low-cost deposition technique (sol–gel). The sensing films were characterized using AFM and SEM as thin film. The transducers are based on an alumina wafer, with Au or Pt interdigital electrodes (IDE) printed onto the alumina surface. The sensor response was recorded upon sensor exposure to different methane concentrations (target gas) under lab conditions (dried target and carrier gas from gas cylinders), in a constant gas flow, with target gas concentrations in the 5–2000 ppm domain and a direct current (DC) applied to the IDE as sensor operating voltage. Humidity and cross-sensitivity (CO_2_) measurements were performed, along with sensor stability measurements, to better characterize the obtained sensors. The obtained results emphasize *good 3-S* sensor parameters (sensitivity, partial selectivity and stability) and also short response time and complete sensor recovery, completed by a low working temperature (220 °C), which are key factors for further development of a new commercial chemiresistor for methane detection.

## 1. Introduction

Methane (CH_4_) is a gas with a major greenhouse effect, being predominantly present in the agricultural areas of the planet. It can accumulate gradually, up to explosive concentrations (50,000 ppm or 5% volume), its formation being particularly favored by warm climates and humidity. Human activities that emit methane include leaks from natural gas systems and the existence of landfills on the outskirts of human settlements. In industry, methane is emitted during LPG refining or from mining activities (coal). Methane is also emitted from natural sources, such as natural wet areas (swamps). Natural processes in the soil and chemical reactions in the atmosphere help to remove CH_4_ from the atmosphere. The lifetime of methane in the atmosphere is much shorter than that of carbon dioxide (CO_2_), but CH_4_ is more efficient at capturing radiation than CO_2_. Quantitatively speaking, the comparative impact of CH_4_ is 25 times greater than that of CO_2_ over a period of 100 years [1].

NIOSH (National Institute for Occupational Safety and Health’s) established a maximum limit of 1000 ppm [2], for an exposure time of 8 h at the workplace, so its detection is very important for safety reasons. Detection of methane without special devices is impossible, methane being odorless and colorless.

The development of gas detectors has increased dramatically in the past decades, starting in 1953, when Brattain and Bardeen discovered that when a gas is adsorbed on the surface of a semiconductor, a change in the electrical conductance of this material occurs [3]. In 1968, Taguchi released the first commercially available gas sensor for the detection of hydrocarbons [4]. Since then, gas sensors, having the advantage of being reduced in size [5] and also cheap devices that can be mass-produced, were used to monitor environmental pollution, obtain global contamination maps [6], monitordomestic safety, ensure public security, monitor automotive safety, monitor air quality and more recently, make medical diagnoses, such as exhaled breath analysis [7]. Gas detectors have been fabricated in many different ways (electrochemical and optical approaches), and solid-state gas sensors contain various gas sensing materials (e.g., metal oxides or MOX). For MOX gas sensors, the most widely accepted sensing mechanism can be explained by the resistance change, which is caused by the surface reaction between the target gas and the sensitive material deposited on the surface of the sensor (in this particular case the sensors are named *chemiresistors*), upon sensor exposure to different gaseous atmospheres [7]. *Chemiresistors* based on semiconductor metal oxides with low-costs, easy production, a compact size and simple electronics are the most widely used in gas detection applications, however, MOX-resistive sensors typically operate at high working temperatures, which limits their application as sensitive materials and leads to sensing material instability, increased power consumption and response drifts [6]. The key for obtaining an economically viable sensor is mainly the low-cost of the final product, which implies abundant raw materials for the sensor components and low-cost preparation techniques (sol–gel, hydrothermal, etc.) for the sensing element, combined with sensor working temperatures as low as possible (ideally room temperature). Ideal materials for gas-sensing applications should be characterized by *high 3-S* parameters (sensitivity, selectivity and stability). Other key features are fast response/recovery time [7].

Although CuO and CoO in different combinations were previously used as sensitive materials [8] for different gases (detection for VOC’s, NH_3_, carbon oxides, H_2_S were summarized in ref. [7]), methane detection (in the percent concentrations range) using these oxides was very rarely reported, and usually high-cost preparation techniques are used to obtain the sensing oxides from their precursors (microwave in ref. [8], thermal oxidation in ref. [9].

In ref. [10], methane detection using Cu-doped CoO was reported, and sol–gel was used in the preparation of the sensitive pellets (compressed powders with silver painted electrodes on each pellet side), but no humidity/cross-sensitivity measurements were taken. Moreover, powder pellets imply usage of large quantities of sensitive material, a non-viable element from an economical point of view. Working temperature of the sensor was also high (300 °C), another important disadvantage.

The aim of this paper was to obtain a cheap, stable, highly sensitive and energy-efficient methane chemiresistor, using thin films of cobalt oxide (CoO) and copper oxide (CuO) as sensitive materials, deposited via a low-cost/eco-friendly technique on an own-design alumina transducer, having Au or Pt IDE’s imprinted on the surface, for the purpose of excellent electrical conductivity. The oxides were used in pristine state, and the sensing performance was then evaluated in each separate case to see which sensor performs better for methane detection. Humidity and cross-sensitivity tests (with CO_2_) were also performed to better define sensor characteristics.

## 2. Results and Discussion

### 2.1. Sensor Characterization

**Figure 1:**
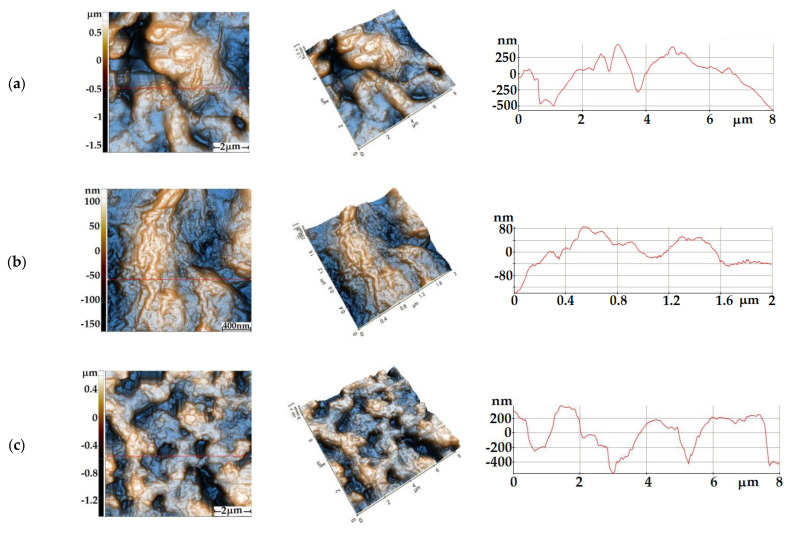

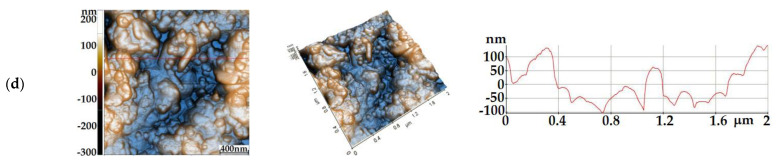
AFM images of the CoO sensitive films at the scale of (8 × 8) µm^2^—(**a**) and (2 × 2) µm^2^—(**b**) and, respectively, of the CuO sensitive films at (8 × 8) µm^2^—(**c**) and (2 × 2) µm^2^—(**d**).

The sensor samples were investigated from a morphological point of view by Atomic Force Microscopy (AFM) after sensing experiments. Figure 1a,b present AFM images of the CoO film deposited by the sol–gel method on alumina substrates configured with Pt interdigitated electrodes (see the Materials and Methods section). The CoO film is characterized by a very high root mean square (RMS) roughness, ~350 nm at the scale of (8 × 8) µm^2^, respectively, a peak-to-valley parameter of ~2477 nm, being the highest values recorded in this series of samples. The surface of the sample is characterized by the presence of protruding massive formations of material (“mountain”-like) but also by random valleys (“pits”)—see the 2D (Figure 1a) and 3D (Figure 1b) AFM images from Figure 1, presented in so-called enhanced contrast view. Scanning a smaller area region, as shown in Figure 1b, suggests that there is a tendency to texturize the film, in the form of “ridges”. At the small scale of (2 × 2) µm^2^, the roughness parameters are significantly reduced, the CoO film having an RMS roughness of ~54 nm, respectively, a peak-to-valley parameter of ~291 nm. Figure 1c,d show AFM images of the CuO film deposited by sol–gel on Pt/alumina interdigitated electrodes. The CuO film shows uniform hills–valleys alternation, as depicted by the profile line from Figure 1c-right, with a level difference of about 900 nm along the selected line. On the entire scanned area of (8 × 8) µm^2^, the global corrugation parameters have the following values: 306 nm for the RMS roughness, respectively, ~2104 nm for the peak-to-valley parameter. Thus, it can be noted that both films prepared by sol–gel are very rough. Scanning a smaller area of (2 × 2) µm^2^ highlights the granular morphology of the CuO film, with a majority population of particles with a diameter in the range of 100–300 nm. The high roughness of the CoO and CuO films evidenced by AFM is an important advantage from the gas sensing point of view, surface roughness being a promoter for the gas adsorption process.

**Figure 2:**
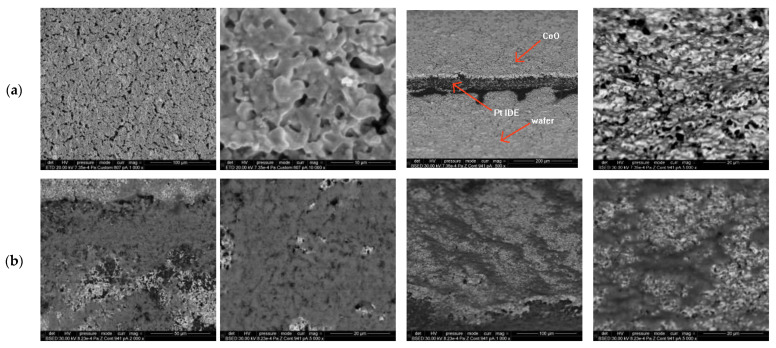
SEM images of: (**a**) CoO sensitive film; (**b**) CuO sensitive film, at different magnification factors; last two images of each group (**a**,**b**) are tilted.

The sensors were then characterized by SEM (Figure 2a,b). The acquired high-RES images confirmed the facts evidenced by AFM investigations: both investigated sensing films having rough surfaces. The morphology of the sensing films deposited by sol–gel adapts very well to the morphology of polycrystalline IDE (Pt/Au) and also to the alumina substrates.

The pores are more visible in SEM images, a network of surface channels being better evidenced in the case of CoO. The third image (tilted) in the Figure 2a group shows the low thickness of the CoO sensitive film (below 1 micron thickness value—the sensitive film may be characterized as *thin*; it appears as a gray coating with a white edge) deposited on a Pt interdigit, which appears to be much thicker than the deposited sensitive layer. As stated before, this increases the accessibility towards the surface adsorption centers for the target gas molecules, increasing the overall sensing capabilities of the oxide film.

### 2.2. Gas Sensing Experiments

**Figure 3:**
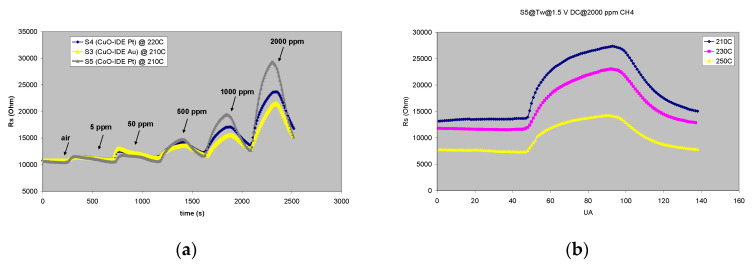
(**a**) The response/recovery of the tested sensors for CH_4_ concentrations in the range of 5–2000 ppm at T_w_ specific to the investigated sensors (210–220 °C); (**b**) response/recovery of the **S5** sensor for 2000 ppm CH_4_ at various working temperatures (T_w_).

All sensing tests were performed *in triplicate* to ensure signal reproducibility. The following sensors were prepared (listed in Table 1) and abbreviated accordingly (as resulted from the synthesis process):

It can be observed that the sensors having CuO sensitive film are available with two IDE types, gold (Au) or platinum (Pt), in order to investigate the influence that the IDE material may have over the sensing experiment. Figure 3a shows the response/recovery characteristics of the sensors presented in Table 1. The sensor with Pt IDE (**S4**) has a slightly higher working temperature—T_w_ (220 °C, comparing with 210 °C for the other two sensors-**S3** and **S5**). Sensor response is comparable when using Pt or Au as IDE (**S4**, **S3**) except in the methane high-concentration range, where cobalt-based sensor-S5 seems to be performing slightly better than the copper-based sensors -**S3**, **S4**, thus supporting the literature findings regarding cobalt oxide [11]. In Figure 3b, a working temperature experiment is depicted for 2000 ppm of CH_4_ injected in the sensing cell equipped with **S5**. It can be observed that with the increasing working temperature, the response/recovery of the sensor decreases; therefore 210 °C is considered T_w_ for this sensor.

Sensor response increases with increasing target gas concentrations (Figure 3a), the highest response being recorded for the concentration of 2000 ppm. All sensors also detect CH_4_ in extremely low concentrations (5 ppm). These detection limits do not represent the lower/higher sensor detection limits, but they are imposed by the technical limitations of the experimental setup, the target gas from the cylinder being diluted by the carrier gas (from 5000 ppm in inert gas, as provided by the gas manufacturer, to a maximum concentration of 2000 ppm or a minimum of 5 ppm, according to the mass-flow controller calibration curves). The response of the sensors is fast (250 s), and the recovery is complete (250 s), making it possible to resume the experiments after the corresponding recovery cycle without sensor replacement.

#### Cross-Sensitivity Tests (Relative Humidity and CO_2_ Measurements)

To better characterize the sensors, cross-sensitivity tests were performed. Thus, the sensors were exposed firstly to a humid atmosphere containing a standard 52% relative humidity (52% RH). Secondly, the sensors were exposed to CO_2_, having concentrations in the 5000–20,000 ppm range, limits imposed by the MFC system in the experimental setup, as stated before. Both these analytes usually accompany methane in the atmospheric environment, being also the main products resulting from the burning of methane when used as gas fuel. The reaction that stands as the basis for the methane principle of detection occurs with an electrical resistance variation, which means there is a change in the charge carrier concentration detected by the measuring equipment (RLC bridge). This mechanism is proposed by Shaalan et al. [8].

(1) $$CH_{4}+4O_{\left(ads\right)}{}^{-}\to CO_{2}{}_{\left(gas\right)}+2H_{2}O_{\left(gas\right)}+4e^{-}$$

**Figure 4:**
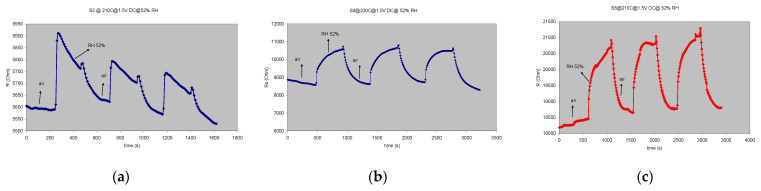
The response/recovery characteristics for 3 successive injections of 52% RH at corresponding T_w_: (**a**) **S3** sensor, T_w_ = 210 °C; (**b**) **S4** sensor T_w_ = 220 °C; (**c**) **S5** sensor, T_w_ = 210 °C.

**Figure 5:**
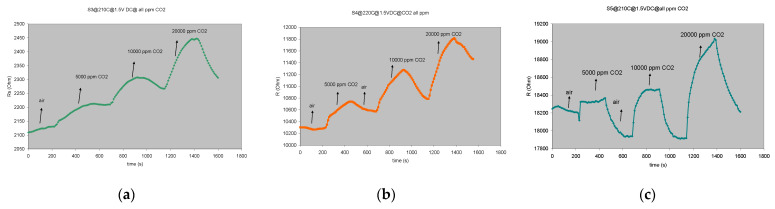
The response/recovery characteristics for 3 successive injections of CO_2_ (5000–20,000 ppm) at corresponding T_w_: (**a**) **S3** sensor, T_w_ = 210 °C; (**b**) **S4** sensor T_w_ = 220 °C; (**c**) **S5** sensor, T_w_ = 210 °C.

The increasing resistance upon p-type sensitive materials (CuO or CoO) exposure to CH_4_ is explained in the mentioned reference as follows: “firstly, the gas reacts with adsorbed negative oxygen ions on the surface, leading to electron injection into p-type oxide. Secondly, this injected electron recombines with a hole in the oxide, reducing its positive free carriers, thus an increase in sensor electrical resistance occurs”. The influence of humidity on the gas sensing properties of metal-oxide-based devices has been extensively investigated, particularly for tin oxide SnO_2_ but is still not comprehensively understood to date [12]. The sensor response to humidity and CO_2_ is shown in Figure 4 and Figure 5.

**Figure 6:**
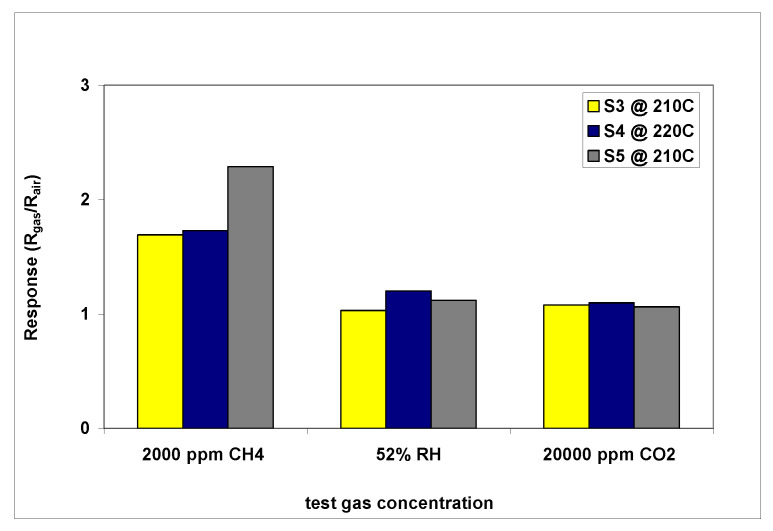
**S3**, **S4**, **S5** cross-response for different tested target gas concentrations.

Figure 6 shows the cross-response of the investigated sensors for the main target gas and the interfering species (resulting from the oxidation reaction that takes place on the surface of the sensor), products which may affect sensor response. It can be seen that for all the investigated sensors responses to 2000 ppm, CH_4_ is almost twice the response to the other interfering species (52% RH and 20,000 ppm CO_2_). The best selectivity is recorded again for the **S5** cobalt-based sensor, where response for CH_4_ is more than double compared with its response towards CO_2_ and humidity. This particular sensor may be considered as *partially selective* for methane.

**Figure 7:**
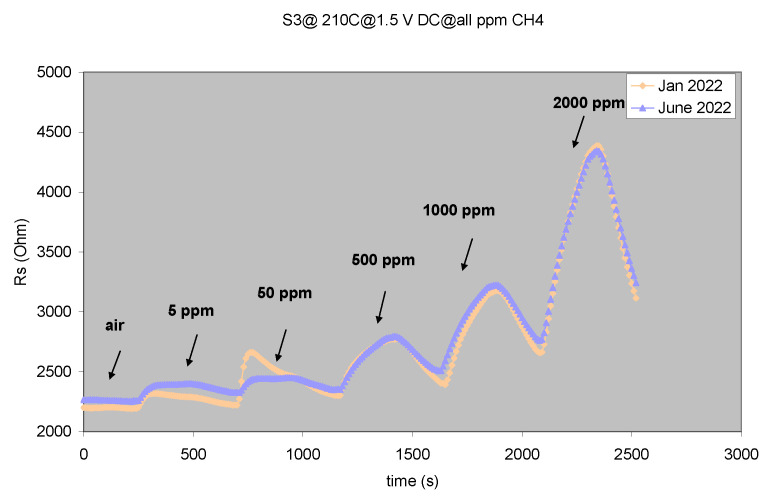
**S3** sensor stability test (6-month period).

As seen in Figure 7, the sensor is stable over a 6-month period between tests, the response to methane in identical experimental conditions being virtually unchanged. Taking into account the results presented in Figure 4, Figure 5, Figure 6 and Figure 7, we can state that the sensors meet the *3-S* parameter requirements: sensitivity, selectivity (partial, for the **S5** sensor sample) and stability, completed by a relatively low working temperature and also short response and full sensor recovery characteristics.

## 3. Conclusions

Sensors with CoO and CuO (MOX based *chemiresistors*) sensitive films have been prepared and characterized. The surface of the sensitive oxide has a high-roughness degree and a large network of channels and pores, as evidenced by AFM and SEM measurements. The films are thin (below 1 micron thick, as shown by SEM). All these characteristics promote gas sensing.

The prepared sensors are stable, partially selective and capable of detecting low concentrations of methane (5 ppm), with fast response (250 s) and a full recovery (250 s). Response for interfering species was recorded (CO_2_ and humidity), but it was relatively low (about 50% from sensors response to methane). Better selectivity was recorded for the cobalt-based sensor towards high methane concentrations. A detection mechanism was formulated in agreement with literature findings. All the investigated sensors are energy-efficient, being characterized by a relatively low working temperature (max. 220 °C). Amongst the investigated sensors, the CoO-based *chemiresistors* are characterized by higher *3-S* parameters (sensitivity, selectivity and stability) compared to the CuO based *chemiresistors*, which place them in a favorable position for further development of a new commercial methane detection MOX based chemiresistor.

## 4. Materials and Methods

Thick sensitive films were obtained using the sol–gel spinning method (1000 rotations/min). As precursors, the basic carbonates of the respective metals were used Cu(CO_3_)_2_Cu(OH)_2_ for CuO and Co(CO_3_)Co(OH)_2_ for CoO.

The deposited film was stabilized by heating it at 400 °C for 10 min.

**Figure 8:**
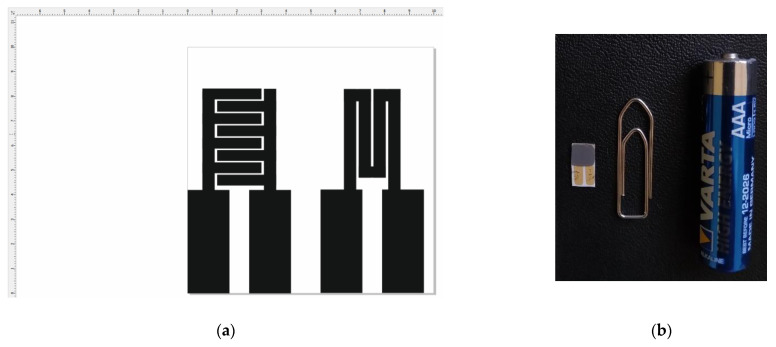
Alumina transducer prototypes for the obtained methane sensors: (**a**) platinum or gold IDE and platinum heater circuit; (**b**) actual **S3** sensor sample compared with various objects for sizing purposes.

Own-design alumina transducers were used, with the following dimensions: 5 × 10 × 0.6 mm (Figure 8). The transducers contained Pt or Au IDE’s on one side and a Pt heater on the opposite side of the transducer. They were imprinted on the alumina surface using serigraphy-based technology.

All sensor measurements were performed under laboratory conditions using dry, high purity gases (5.0), purchased from specialized gas-suppliers (SIAD Romania). The gas concentrations in the cylinders were: 5000 ppm CH_4_ and 50,000 ppm for CO_2_ (both in inert gas), as stated by the certification labels. The operating voltage of the sensor was set at 1.5 V direct current (DC), the tested working temperatures (T_w_) were in the range situated between room temperature and 220 °C (specific for each sensor used), and the sensing experiments were carried out in a continuous flow of gas (maximum 180 mL/min).

**Figure 9:**
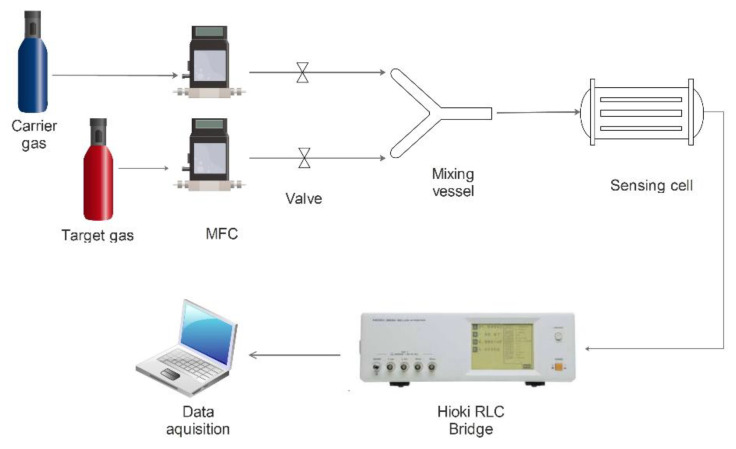
Schematic representation of the gas sensing experimental setup.

Two separate gas lines were used for the sensing experiments, one ensuring the flow of the carrier gas (dry air), the other ensuring the flow of the target gas. The target gas concentrations were achieved using a calibrated system of mass-flow controllers (MFC). The two separate gas flows were mixed inside a special glass vessel, shown in the scheme of the experimental installation (Figure 9), using an on-off valve system, thus diluting the target gas with the carrier gas.

The gas route continues to an own-design sensor cell, which contains the investigated sensor. In the sensor cell, a chemical reaction takes place on the surface of the sensor, which leads to a change in its electrical resistance, a variation recorded by the Hioki 3522-50 RLC bridge connected to the sensing cell. This bridge uses a “custom-made” acquisition software [13,14,15], based on the Labview platform, developed by our group. Thus, the analog signal taken from the chemiresistor was converted into a digital signal using a GPIB interface connected to the output of the RLC bridge. The resulting digital signal is then transformed into an *xy* graph using the data acquisitioning computer [16,17,18].

The sensors were characterized (after sensing experiments) by AFM and SEM to observe surface morphology for the sensing CuO and Co O films.

AFM measurements were carried out in non-contact mode [19] with XE-100 (Park Systems), using sharp tips (NCHR from Nanosensors), having less than 8 nm tip radius, ~125 µm length, ~30 µm mean width, thickness ~4 µm, ~42 N/m force constant and ~330 kHz resonance frequency. The XEI (v.1.8.0) image processing program developed by Park Systems was used for displaying the images and subsequent statistical data analysis.

The microstructure of the samples was investigated by SEM using a high-resolution microscope (FEI, Quanta 3D FEG). The analyses were performed in high vacuum mode at high accelerating voltages (30 kV), and the sensors were analyzed directly [19,20,21,22] (samples were immobilized on a double-sided carbon tape, without coating).

## Figures and Tables

**Table 1:** The investigated sensors and their composition.

Sensor Abbreviation	Sensitive Film	Transducer (IDE/Wafer)
**S3**	**CuO**	**Au**/Al_2_O_3_
**S4**	**CuO**	**Pt**/Al_2_O_3_
**S5**	**CoO**	**Pt**/Al_2_O_3_

## Data Availability

The datasets generated during and/or analyzed during the current study are available from the corresponding author on reasonable request.
